# An Account of Some Symptoms of Fever, and of the Means of Removing Them

**Published:** 1783

**Authors:** 


### III. *An Account of ſome ſymptoms of fever, and of the means of removing them. By* Thomas Sanden, M. D. *Phyſician at Chicheſter.*

IT has been a matter of doubt with ſome, whether ſuch a diſeaſe as *ſynocha* is defined to be, ever exiſts, or, in other words, whether there is ſuch a thing as a purely inflammatory fever accompanied with a topical inflammatory affection. I do not pretend to diſcuſs this general queſtion, and ſhall only obſerve that there may be ſometimes in fevers a phlogiſtic diatheſis, which is *latent*, or at leaſt which does not betray itſelf by the uſual and more evident ſymptoms.

The ſpring of the year 1781 was (in this neighbourhood) remarkable for great drought in the beginning, and afterwards for ſudden interchanges of exceſſive heat, with a moderate or rather cold temperature of the air. The ſame ſeaſon was alſo remarkable for this (as I have called it) *latent* phlogiſtic diatheſis. It took place in many diſeaſes, and frequently at the decline of them, when they had ſubſiſted for a while, and much weakened the patient. The marks of it were not a ſtrong, full, hard pulſe, great heat and thirſt, dry ſkin and high-coloured urine, as is uſual; but rather reſtleſſneſs and anxiety, with the abſence of the ſigns of a perfect criſis to the fever; that is, the appetite did not return, the tongue continued foul, and the urine pale and clear. The nature of the complaint became completely manifeſt only in conſequence of the firſt trial of the proper remedy; for on letting blood the ſymptoms were certainly relieved, the pulſe was raiſed, and the extravaſated blood (properly drawn) hardly ever failed to be covered with an inflammatory cruſt. It is not to be wondered at that the intermittent fevers of the above ſeaſon ſhould partake ſtrongly and evidently of this general phlogiſtic tendency; but ſome had it where the marks were obſcure, and where one would little expect to find it. A middle-aged man laboured under an intermittent complaint, which attacked him every other day under the form of a violent head-ach, terminating in a ſweat. Moderate evacuations by blood-letting, purging, and vomiting were uſed: the diſeaſe, however, ſoon changed to a quotidian type. After no great number of revolutions the ſymptoms were by degrees got to ſuch a height, that the patient ſoon after he was ſeized with his head-ach became delirious, inarticulate in his ſpeech, and almoſt ſenſeleſs. In his ſecond paroxyſm of this kind I ſaw him. The heat of the the ſkin was not greater than natural. The pulſe beat only fifty ſtrokes in a minute, and were very ſtrong and hard. The immediate loſs of a large quantity of blood, a bliſter applied over the whole head, and an active purge, which was taken without delay, removed theſe alarming ſymptoms; they never returned, and the patient entirely recovered his health by the uſe of ſome ſlight antiphlogiſtic remedies and a low diet. I muſt here obſerve, that I had myſelf contributed to the violence of the paroxyſm above deſcribed. Alarmed at the account given me of the preceding paroxyſm, and having my mind filled with I know not what ideas of intermittent fevers aſſuming the malignant forms of lethargy, apoplexy, &c. as deſcribed by Werlhof, Torti, and others, I dreaded nothing more than the recurrence of the fit, and preſcribed the Bark in large quantities, and this remedy was indeed adminiſtered with ſo much diligence, that the patient took not leſs than three ounces of it in ſubſtance in the ſpace of about fourteen hours. This was no doubt a great miſtake, and very injurious to the patient. I ought to have conſidered the genius of the prevailing diſeaſes, and the change which this particular diſeaſe had undergone from a tertian to a quotidian type. Theſe circumſtances ſhould have taught me, that tho' ſome dangerous diſeaſes of the intermittent kind (of which I had been not without experience) were to be cured only by proper remedies, as, *ex. gr.* the bark, given liberally in the intermiſſion, there were others (of which the preſent was an inſtance) which would be ſucceſsfully attacked only in the time of the paroxyſm, and by ſuch remedies as the nature of the paroxyſm itſelf ſeemed to require. Another inſtance of this kind I had occaſion to obſerve in the autumn of the ſame year 1781. This was a remittent tertian, and the moſt remarkable ſymptom in the paroxyſm (which was alſo accompanied with increaſed heat, frequency of the pulſe, &c.) was a hoarſeneſs. This indeed was never intirely off, but it was prodigiouſly increaſed in the paroxyſm, and was attended with a ſenſe of ſome ſtricture of the breaſt. The debility of the patient and the weakneſs of the pulſe were ſuch as to forbid blood-letting. After an emetic two or three times repeated, and a few ſaline draughts, the bark was given, but it rather exaſperated the ſymptoms, which were at length removed by inhaling the ſteam of hot water, the application of a volatile liniment with camphor to the throat, and a few ſimple remedies that ſeemed to have no ſenſible effect but that of looſening the belly.

---

The ufe of cathartics in fevers has been a fubject of great difpute, owing, in part, I believe to the exceffive and injudicious veneration which fome have entertained for the writings of Hippocrates; it being well known, that what this great father of phyfic has left on the fubject of thefe remedies is by no means in favour of their ufe. Among the more celebrated moderns, Werlhof, De Haen, and perhaps Sydenham, may be efteemed in general as very little inclined to employ cathartics in fevers, while Senac, Cleghorn, and Pringle, as well as many others, lay no fmall ftrefs on the timely and moderate exhibition of thefe remedies.

Prejudice, which too often ufurps the name and precludes the benefits of experience, has, as in many others, mingled itfelf alfo in this queftion. I fpeak not of M. de Haen, whofe known prepoffeffions in fome degree difcredit his otherwife valuable writings; but the candid and fagacious Werlhof evidently is not free from bias. Witnefs the following paffage, "Vaniffimum effe conftat, quod aliqui prætexunt, "prifcos medicos, cum regulas de purgatione "fcriberent, non nifi fortiffima pharmaca nota

"habuiffe,

"habuiſſe, leniorum ignaros." *Obſervat. de Febrib.* ſect. 1. § vii. In anſwer to this, little more ſeems neceſſary than an enumeration of the purging remedies moſt frequently uſed by the antients. We have beſides ſufficient evidence that Hippocrates in particular, and ſome of his diſciples, were very cautious in the uſe of theſe remedies, and that they had reaſon for being ſo, as we learn from Celſus (lib. 2. c. xii.) and from the author of the Treatiſe Περι φαρμακων (§ iv.) inſerted among the works of Hippocrates. It will be allowed, indeed, that the ancients did, on ſome occaſions, uſe laxatives of no draſtic or violent operation; yet we have reaſon to believe that theſe laxatives were much leſs generally applied than the rougher medicines, that they might with propriety be conſidered rather as articles of diet, and that for the moſt part they were of weak and uncertain effect.

But the diſcuſſion of theſe general queſtions is quite foreign from my purpoſe. Two particular caſes of fever occur to my recollection, which ſtrongly illuſtrate the neceſſity of cleanſing the primæ viæ either in the beginning, or in the more advanced periods of the diſeaſe. The firſt was the caſe of a gentleman, who in the beginning of a remittent fever that attacked him towards towards the decline of a very hot ſummer, had as unequal and irregular a pulſe as I remember ever to have felt. As there was no other threatening ſymptom, I was very little alarmed at this, but truſted to the operation of a few moderately large doſes of James's powder, which vomiting and purging the patient ſmartly, reduced the pulſe to a perfectly even tenor. The other caſe was of a lady, who in November laſt was ſeized with a fever about three weeks after lying-in. Her child had died, and ſhe was ſuppoſed to have ſuffered much from a concealed and ſtifled grief. The more urgent ſymptoms in this caſe were, pain in the back part of the head; ſickneſs at ſtomach (to remove which ſeveral emetics had been taken, and always with more or leſs relief); a frequent ſpitting of frothy ſaliva; a total want of natural ſleep; a very remarkable proſtration of ſtrength, and much deſpondency and dejection of a mind naturally calm and reſolute. The mean frequency of the pulſe was from 118 to 126, with conſiderable ſtrength in the ſtroke of the artery. About the thirteenth day from the attack, as nearly as could be aſcertained in a diſeaſe, the beginning of which was not very diſtinctly marked, the patient was ſeized in the evening with a faintneſs, which alarmed the the attendants very much; this lafted for an hour or two, and returned in a lefs degree, and at intervals through the night. The next morning the faintnefs came on again, and in a ftill more alarming manner; the fits lafting longer, being more violent, and accompanied with coldnefs of the face and extremities, a clammy fweat, &c. The patient had been a few hours in this fituation when I faw her. It was with confiderable difficulty that fhe could articulate her words, and tell me that fhe felt the hand of death upon her, and that fhe had given up all hopes of recovery. Her countenance was funk and her lips pale. Feeling her pulfe, I was furprized to find that with a frequency amounting to 136 beats in a minute, they had very great ftrength and tenfion, and I had afterwards occafion to remark that the greater the patient's faintnefs appeared to be, the ftronger and firmer were the ftrokes of the artery. This encouraged me mightily. In the mean time the patient paffed a ftool attended with the fenfation of great heat and uneafinefs; and the frothy fpitting, above mentioned, ftill continued fo frequent as to incommode the patient in the very depreffed ftate fhe was in. All thefe circumftances contributed to determine my future proceedings.

Early

Early in the evening the patient took ſix grains of James's powder, and another equal doſe four hours afterwards. This quantity of the antimonial, with about an ounce and an half of vinum ipecacoanhæ given *partitis vicibus* through the night produced little evacuation by vomiting, but an almoſt conſtant nauſea, and ſeveral copious, dark-coloured and extremely fœtid ſtools. During the whole of this operation the patient felt not the leaſt ſymptom of returning faintneſs, and the frequency of the pulſe (notwithſtanding the neceſſary exertions) was rather leſſened than encreaſed. In order to quiet the ineffectual retching, which continued to fatigue the patient till the next morning was pretty far advanced, a few drops of tinct. thebaic. were given. Theſe, after a while, procured an hour or two of refreſhing ſleep. A copious perſpiration ſucceeded, and laſted through the greater part of the day. In the evening the pulſe were reduced to 108 ſtrokes in a minute, and were ſoft and free. From this time the patient began to get better, though her recovery was very gradual and ſlow; and it is to be remarked, that though attempts were ſeveral times made to give the bark in different forms, this was never with any good effect, but generally with an unfavourable one, till ſeveral weeks had elapfed after that period of the difeafe, which was attended with the appearances above mentioned.

I fhall conclude this paper with a remark, which, though not immediately connected with what goes before, may be thought not entirely foreign from it. The connection between the ftate of the inteftinal canal and the external mufcular parts is properly noticed by the late Dr. Akenfide in his Treatife *de Dyfenteriâ*, p. 22. I have obferved, that pains of the abdomen in young fubjects are often attended with pain, and particularly with extreme forenefs of the legs and thighs. A boy five years old was feized with vomiting and pain of the ftomach and belly, and foon became very feverifh. At length the pain fixed itfelf in the right hypochondriac region, a little above the fpine of the ilium, and was attended with very great lamenefs of the right thigh, infomuch that fome motions of that limb, particularly in the way of extenfion, produced intolerable pain. Thefe circumftances made me apprehenfive of inflammation and impending fuppuration of fome of the internal mufcular parts of the pelvis. Moft of the ufual remedies were tried on this fuppofition, but to no purpofe: at laft a brifk purge produced a copious ous evacuation of a matter resembling yeast, to the entire relief of the patient, who began from that time to recover, and soon regained, without using any other remedy, the perfect use of the affected limb.

*Chichester,*

*July* 25, 1783.

